# What is the “cost” of reducing adverse pregnancy outcomes in patients with gestational diabetes mellitus - risk factors for perinatal complications in a retrospective cohort of pregnant women with GDM

**DOI:** 10.1186/s12884-022-04980-w

**Published:** 2022-08-19

**Authors:** Luiza Oleszczuk-Modzelewska, Aneta Malinowska-Polubiec, Ewa Romejko-Wolniewicz, Agnieszka Zawiejska, Krzysztof Czajkowski

**Affiliations:** 1 2nd Department of Obstetrics and Gynaecology, Medical University of Warsaw, 2 Karowa St, 00-315 Warsaw, Poland; 2 Department of Medical Simulation, Chair of Medical Education, Poznan University of Medical Sciences, 41 Jackowskiego St, 60-512 Poznan, Poland

**Keywords:** Gestational diabetes mellitus, Risk factors, Obstetric outcomes

## Abstract

**Background:**

Gestational diabetes mellitus (GDM) is a frequent pregnancy complication, affecting the maternal and neonatal health. The new diagnostic strategy for GDM, proposed by the International Association of Diabetes and Pregnancy Study Groups in 2010 and World Health Organization in 2013, raised hope to reduce perinatal complications. The purpose of the study was to compare risk factors influencing maternal and foetal outcomes in a group of pregnant women diagnosed with GDM, and in a group of pregnant women without GDM, regardless of the adopted diagnostic criteria. Also, the aim of the study was to evaluate the impact of risk factors on perinatal results and the “cost” of reducing adverse pregnancy outcomes in patients with GDM.

**Methods:**

It was a retrospective study based on the analysis of births given after 37 weeks of pregnancy at the 2nd Department of Obstetrics and Gynaecology, Warsaw Medical University during the years 2013 to 2015. All pregnant women had a 75 g OGTT between the 24th and 28th weeks of pregnancy. The study compared risk factors for perinatal complications in 285 GDM patients and in 202 randomly selected women without GDM. The impact of selected risk factors on perinatal outcomes was analysed.

**Results:**

Both the diagnosis of GDM and maternal BMI prior to pregnancy, significantly modified the risk of excessive and insufficient weight gain during pregnancy. The parameters significantly influencing the risk of the composite adverse maternal outcome were the maternal abdominal circumference [OR: 1.08 (1.04; 1.11)] and multiparity, which reduced the risk by almost half [OR: 0.47 (0.30; 0.75)]. The maternal abdominal circumference before the delivery was a strong factor correlating with the occurrence of perinatal complications in both the mother and the foetus in the entire cohort. A circumference over 100 cm increased the risk of at least one maternal complication (increased blood loss, soft tissue injury, pre-eclampsia) by almost 40% (OR 1.38, *p* < 0.001).

**Conclusions:**

No differences were found in maternal and foetal outcomes in GDM and non-GDM women except gestational weight gain below Institute of Medicine recommendations. The only “cost” of reducing adverse pregnancy outcomes in GDM patients seems to be lowering gestational weight gain, the future impact of which on GDM pregnant population should be assessed. The maternal abdominal circumference measured before delivery not the severity of carbohydrate intolerance, remained the main predictor for significant perinatal complications.

## Background

Gestational diabetes mellitus (GDM) includes all types of impaired glucose tolerance that are first experienced or diagnosed during pregnancy. Maternal complications of GDM include pregnancy-induced hypertension, pre-eclampsia, the need to induce labour and the necessity to deliver the baby by caesarean section. It has been proven that in the future, these women are more likely to develop diabetes, cardiovascular diseases and metabolic syndrome [1, 2]. Foetuses of patients with gestational diabetes are more frequently diagnosed with large for gestational age (LGA), macrosomia and a higher percentage of perinatal injuries. New-borns of mothers with GDM are at risk of developing respiratory disorders, hypoglycaemia and hyperbilirubinemia. It is believed that in the future, these children will more often suffer from diabetes, obesity, hypertension and metabolic syndrome [3, 4].

For several years, a progressive increase in the percentage of diabetes cases diagnosed in the world, including in pregnant women, has been observed. The International Diabetes Federation (IDF) reported that 16,7% (21.1 million) of live births to women in 2021 had some form of hyperglycaemia in pregnancy. Of these, 80.3% were due to gestational diabetes mellitus [5]. According to the database of the National Health Fund, in Poland, the proportion of women with GDM amounted to 4.7% in 2010 and increased to 7.5% in 2012 [6]. Getahun et al. found that in a period of over 15 years (1989–2004), the number of patients with GDM in the American population increased by 122% [7]. Despite the growing trend of diabetes in pregnancy, there is still no consensus among leading diabetes societies regarding screening for GDM. In 2010, International Association of Diabetes and Pregnancy Study Groups (IADPSG), and in 2013 the World Health Organization (WHO) proposed changing the existing criteria for the diagnosis of GDM [8, 9]. In the new diagnostic strategy for gestational diabetes, based on results of the Hyperglycaemia and Adverse Pregnancy Outcome (HAPO) Study from 2008, the diagnostic criteria for this disease were associated with the risk of neonatal complications and not with the long-term risk of developing type 2 diabetes in the mother, as was the case so far [10]. In Poland, they came into force in 2014.

The new recommendations raised hope for a standardization of the system for diagnosing diabetes in pregnancy and thus a reduction in perinatal complications, the percentage of which is inconclusive [11–15].

The purpose of the study was to compare risk factors influencing maternal and foetal outcomes in a group of pregnant women diagnosed with gestational diabetes mellitus, and in a group of pregnant women without GDM, regardless of the adopted diagnostic criteria. Also, the aim of the study was to evaluate the impact of risk factors on perinatal results and the “cost” of reducing adverse pregnancy outcomes in patients with GDM.

## Methods

**Fig. 1:**
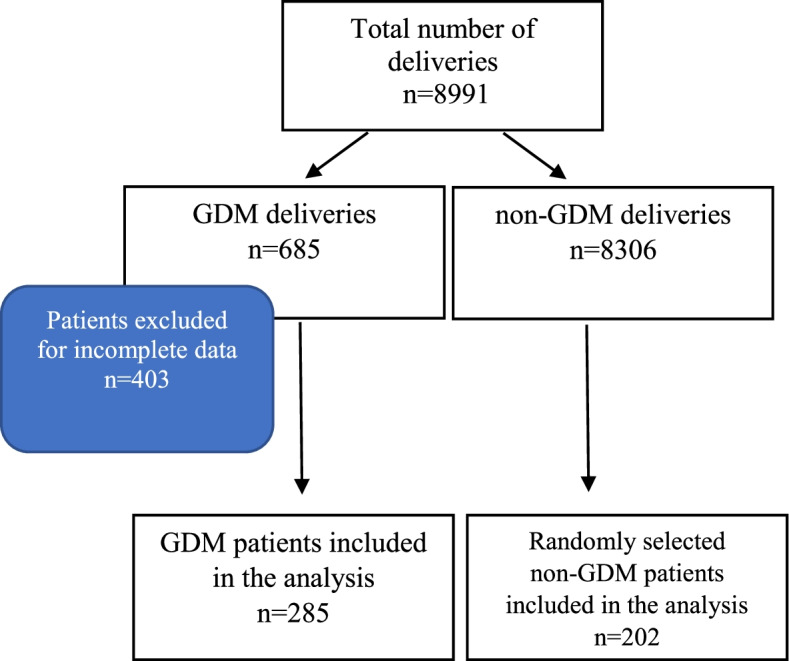
Flow diagram of the study

This was a retrospective study based on the analysis of births that occurred at the 2nd Department of Obstetrics and Gynaecology, Warsaw Medical University during the period 1st January 2013 – 31st December 2015. During this time there were 8991 deliveries, of which GDM patients accounted for 685. The analysis included patients who gave birth after 37 weeks of pregnancy. All pregnant women had an oral glucose tolerance test (OGTT) of 75 g between the 24th and 28th weeks of pregnancy. The following patients were excluded from the analysis: patients with multiple pregnancy, patients with pre-gestational diabetes, gestational diabetes diagnosed on the basis of abnormal fasting glucose results during early pregnancy, random glycaemia, or OGTT of 75 g performed before the 24th or after the 28th weeks of pregnancy, and those with incomplete results of the three-point OGTT. The group of 285 pregnant women with diagnosis of GDM and complete data entered the study. At the same time, 202 pregnant women without GDM were randomly selected as controls. In total, 487 patients were included into the study. The size of the cohort was calculated to get at least 90% statistical power for the most commonly reported foetal and maternal complications in GDM: neonatal birth weight > 4000 g, LGA, and hyperbilirubinemia, preeclampsia, and postpartum haemorrhage. The study flow diagram is presented in Fig. 1.

The 2011 criteria for diagnosing GDM in Poland using a 75-g OGTT required at least one of the following glucose values: fasting ≥100 mg/dL, 1 hr. ≥180 mg/dL, or 2 hrs ≥140 mg/dL. Meanwhile, the diagnostic criteria adopted in 2014, according to IADPSG and WHO, required at least one of the following results of the 75-g OGTT: fasting ≥92 mg/dL, 1 hr. ≥180 mg/dL, or 2 hrs ≥153 mg/dL. Due to the update of diagnostic criteria during the study period we decided to incorporate all patients who met any set of standards.

Data were retrieved from the database of the referral center. All diabetic women were advised to attend a medical consultation with an obstetrician. Diabetes-specific counseling included an explanation of possible risks to the mother and the fetus related to pregnancy and the methods to reduce possible complications, including glycemic goals, lifestyle management, and medical nutrition therapy. Fasting and postprandial blood glucose self-monitoring was recommended in GDM patients to achieve metabolic control. Glucose targets recommended by the Polish Society of Gynecologists and Obstetricians (similar to the targets recommended by the American College of Obstetricians and Gynecologists and the American Diabetes Association) were as follows: fasting < 95 mg/dL (5.3 mmol/l), and either 1 hr. postprandial < 140 mg/dL (7.8 mmol/l) or 2 hrs postprandial < 120 mg/dl (6,7 mmol/l). If glycaemic control was not satisfactory with nonpharmacological treatment, insulin therapy was introduced [16, 17].

The study compared patients with GDM to pregnant women without GDM. The risk factors for perinatal complications were the following: maternal age, maternal height and body mass index (BMI) prior to pregnancy, multiparity (at least one child delivered), history of GDM, history of neonatal birth weight (BW) > 4000 g, birth weight of the largest child from previous pregnancy/pregnancies, gestational age at GDM diagnosis/at testing, fasting blood glucose level, 1 hr. and 2 hrs blood glucose level of the 75-g OGTT, diagnosis of diabetes during the current pregnancy, gestational weight gain (GWG), the maternal abdominal circumference measured before delivery at the level of the navel, the need to implement insulin therapy to maintain normoglycaemia and female sex of the new-born (Table 1).

**Table 1:** Potential risk factors for maternal and foetal outcomes in the study group

	Women with GDM *N = 285 (58.5%)*	Women without GDM *N = 202 (41.5%)*	*P*
Age [years]	32.4 ± 4.7	31.1 ± 4.1	< 0.001
Maternal height [cm]	164.29 ± 5.7	167.96 ± 5.5	0.59
Pre-pregnancy BMI [kg/m^2^]	24.6 ± 4.6	22.7 ± 3.6	< 0.001
Pre-pregnancy BMI ≥ 25.0 kg/m^2^ [%]	37.7%	25.7%	0.006
Pre-pregnancy BMI ≥ 30.0 kg/m^2^ [%]	13.5%	3.0%	< 0.001
Multiparity [%]	61.8%	49.5%	0.024
History of GDM [% of these with the history of at least one delivery]	18.8%	0.0%	< 0.001
History of BW > 4000 g [% of these with the history of at least one delivery]	16.3%	10.6%	0.249
BW of the largest child from previous pregnancy/pregnancies [g]	3436 ± 629.53	3321.8 ± 617.9	0.86
Gestational age at diagnosis/at testing [weeks]	25.4 ± 1.5	25.2 ± 1.5	0.055
75 g OGTT fasting [mg/dL]	87.1 ± 11.7	77.5 ± 6.5	< 0.001
75 g OGTT 1 hr. [mg/dL]	166.5 ± 28.2	117.3 ± 25.5	< 0.001
75 g OGTT 2 hrs [mg/dL]	144.2 ± 23.3	100.0 ± 19.1	< 0.001
GDM according to 2011 or 2014 criteria [%]	61.6%	–	
Gestational weight gain [kg]	10.7 ± 5.8	14.5 ± 5.1	< 0.001
Maternal abdominal circumference [cm]	103.3 ± 8.7	103.0 ± 6.9	0.969
Insulin therapy necessity [%]	15.4%	–	
Female foetus [%]	48.4%	54.0%	0.223

*GDM* Gestational Diabetes Mellitus, *BMI* Body Mass index, *BW* Birth Weight, *OGTT* Oral Glucose Tolerance Test

The impact of the aforementioned factors on perinatal outcomes was analysed. The maternal outcomes included in the analysis were as follows: gestational weight gain (GWG) with respect to Institute of Medicine (IOM) recommendations, incidence of pregnancy-induced hypertension or pre-eclampsia, mode of delivery (caesarean section/forceps or vacuum/ spontaneous), the necessity to perform a caesarean section due to foetal indications, incidence of intrapartum maternal injury, intrapartum haemorrhage (blood loss more than 500 mL), and composite adverse maternal outcome (including intrapartum maternal injury or haemorrhage). The following neonatal outcomes were analysed: the new-born birth weight, including the percentage of children with macrosomia (birth weight > 4000 g), the new-born ponderal index, the incidence of hypoglycaemia or hyperbilirubinemia (treated with phototherapy during the first 3 days of the new-borns’ life), the percentage of children with congenital anomalies, intrapartum neonatal injury (including clavicle fracture, brachial palsy, intraventricular haemorrhage) and rare neonatal complications with the occurrence below 5% (including intrapartum neonatal injury or hypoglycaemia) (Table 2).

**Table 2:** Maternal and foetal outcomes in the study group

	Women with GDM *N = 285 (58.5%)*	Women without GDM *N = 202 (41.5%)*	*P*
Gestational weight gain [kg]	10.7 ± 5.8	14.5 ± 5.1	< 0,001
GWG above IOM recommendations [%]	24.2%	47.5%	< 0.001
GWG below IOM recommendations [%]	45.6%	20.3%	< 0.001
Pregnancy induced hypertension/ preeclampsia [%]	6.0%	4.5%	0.543
Mode of the delivery: CS/Forceps or VE/ Spontaneous	31.9%/3.5%/64.6%	33.7%/5.0%/60.4%	0.599
Emergency CS due to foetal conditions^a^	26.4%	16.2%	0.06
Intrapartum maternal injury	6.1%	8.0%	0.468
Intrapartum haemorrhage	32.0%	27.2%	0.271
Composite adverse maternal outcome	37.9%	35.1%	0.568
BW [g]	3412 ± 438	3420 ± 428	0.677
BW > 4000 g	9.1	8.9	1.00
Ponderal index [g/cm^3^]	2.20 ± 0.26	2.18 ± 0.24	0.18
Neonatal hypoglycaemia	4.9%	–	–
Neonatal hyperbilirubinemia	21.1%	17.3%	0.413
Foetal congenital malformation	7.7%	5.9%	0.477
Intrapartum neonatal injury	3.2%	5.4%	0.250
Rare adverse neonatal outcome	8.1%	5.4%	0.285

*GDM* Gestational Diabetes Mellitus, *GWG* Gestational Weight Gain, *IOM* The Institute of Medicine, *CS* Caesarean section, *VE* Vacuum Extraction, *BW*:Birth Weight

^a^percentage of cases out of all caesarean sections

We used SPSS 14.0 for Windows (SPSS Inc. Chicago, USA) and MedCalc 19.0 (MedCalc Software, Mariakerke, Belgium) to perform statistical analysis.

Descriptive results are expressed as the mean ± standard deviation or median and interquartile range. Categorical variables are expressed as percentages.

Multivariate logistic regression model was used to identify predictors for dichotomous adverse maternal and foetal outcomes in the pooled analysis of the entire group (patients with GDM and normoglycemic controls). Variables that correlated with the outcomes with a *p* < 0.1 in the bivariate analysis were included in the models. The results are presented as adjusted odds ratios (aORs) and standardized coefficients with 95% confidence intervals (95% CIs) (Table 3). Predictors for continuous variables were identified using multiple linear regression models, with gestational weight gain or birth weight as dependent variables. All variables that showed a bivariate correlation with a *p* < 0.1 with these outcomes were entered in the models as independent variables. A *p* < 0.05 was considered statistically significant in the multiple regression analysis. The results are presented as unstandardized and standardized coefficients with 95% confidence intervals (Table 4).

**Table 3:** Predictors of maternal and foetal outcomes in the study group – analysis of multivariate logistic regression models

The outcome	Variables in the model	exp(B) (95%CI)^*^	Standardized coefficients (95% CI)	*P*	*R*^*2*^ for the model
GWG above recommendations	75 g OGTT fasting [mg/dL]	1.03 (1.00; 1.05)	0.000 (0,000; 0,001)	0.023	0.190
	75 g OGTT 2 hrs [mg/dL]	0.99 (0.98; 0.99)	0.000 (0.000; 0.000)	0.022	
	Pre-pregnancy BMI [kg/m^2^]	1.12 (1.06; 1.18)	0.003 (0.002; 0.004)	< 0.001	
	GDM yes/no	0.31 (0.16; 0.57)	−0.374 (− 0.571; − 0,178)	< 0.001	
	Female neonate yes/ no	0.56 (0.37; 0.85)	−0.121 (− 0.207; − 0.034)	0.006	
GWG below recommendations	Pre-pregnancy BMI [kg/m^2^]	0.90 (0.85; 0.95)	−0.003 (− 0.004; − 0.001)	< 0.001	0.162
	GDM yes/no	4.44 (2.85; 6.92)	0.337 (0.237; 0,437)	< 0.001	
	Female neonate yes/no	1.65 (1.10; 2.47)	0,103 (0.020; 0.185)	0.015	
Preeclampsia and/or gestational hypertension	Pre-pregnancy BMI [kg/m^2^]	1.15 (1.06; 1.3)	0.006 (0.002; 0.10)	0.001	0.123
	Multiparity yes/no	0.24 (0.08; 0.73)	−0.82 (−1.47; − 0.18)	0.012	
Composite adverse maternal outcome	Maternal abdominal circumference [cm]	1.08 (1.04; 1.11)	0.001 (0.001; 0.002)	< 0.001	0.123
	Multiparity yes/no	0.47 (0.30; 0.75)	−0.176 (− 0.284; − 0.067)	0.001	
Emergency CS due to foetal conditions	Multiparity yes/no	0.30 (0.13; 0.70)	−0.510 (− 0.877; − 0.152)	0.005	0.133
	Maternal height [cm]	0.91 (0.85; 0.98)	−0.030 (− 0.005; − 0.001)	0.007	
	Maternal abdominal circumference [cm]	1.08 (1.03; 1.12)	0,002 (0.001; 0.002)	< 0.001	
BW > 4000 g	Maternal abdominal circumference [cm]	1.07 (1.03; 1.11)	0.001 (0.001; 0.002)	0.001	0.183
	Maternal height [cm]	1.07 (1.00; 1.14)	0.002 (0.00; 0.004)	0.036	
	Multiparity yes/no	2.20 (1.03; 4.70)	0.305 (0.012; 0.599)	0.042	
	Female neonate yes/no	0.21 (0.09; 0.53)	−0.705 (−1.118; −0.293)	0.001	
Neonatal hypoglycaemia – *data available only for women with GDM*	Maternal abdominal circumference [cm]	1.12 (1.06; 1.19)	0.004 (0.002; 0.006)	< 0.001	0.193
Neonatal hyperbilirubinemia / phototherapy	Insulin therapy yes/no	2.84 (1.27; 6.35)	0.337 (0.063; 0.611)	0.011	0.128
	Pre-pregnancy BMI [kg/m^2^]	1.07 (1.02; 1.1)	0.002 (0.000; 0.004)	0.002	
Rare adverse neonatal outcome	Maternal age [years]	0.89 (0.82; 0.98)	−0.005 (−0.009; −0.001)	0.013	0.124
	Maternal abdominal circumference [cm]	1.08 (1.03; 1.13)	0.002 (0.001; 0.003)	< 0.001	
	Maternal height [cm]	0.90 (0.84; 0.97)	−0.004 (−0.006; − 0.001)	0.004	

*CI* Confidence interval, *OGTT* Oral Glucose Tolerance Test, *BMI* Body Mass Index, *GWG* Gestational Weight Gain, *BW* Birth Weight, *GDM* Gestational Diabetes Mellitus, *CS* Caesarean Section

**Table 4:** Predictors of maternal and foetal outcomes in the study group – analysis of multiple linear regression models

The outcome	Variables in the model	Unstandardized coefficients B (95%CI)	Standardized coefficients (95%CI)	*P*	*R*^*2*^ for the model
Gestational weight gain [kg]	75 g OGTT 2 hrs [mg/dL]	−0.04 (− 0.060; − 0.016)	−0.20 (− 0.3; − 0.08)	0.001	0.183
	Pre-pregnancy BMI [kg/m^2^]	−0.23 (− 0.35; − 0.12)	−0.17 (− 0.26; − 0.09)	< 0.001	
	GDM yes/no	−2.43 (−3.88; − 0.98)	−0.21 (− 0.34; − 0.08)	0.001	
	Female neonate yes/no	−1.23 (−2.18; − 0.28)	−0.10 (− 0.18; − 0.02)	0.011	
	75 g OGTT fasting [mg/dL]	0.07 (0.02; 0.12)	0.14 (0.04; 0.24)	0.005	
	Multiparity yes/no	−1.15 (−2.11; −0.19)	−0.1 (− 0.18; − 0.02)	0.015	
Birth weight – *for the whole cohort*	Maternal abdominal circumference [cm]	12.1 (6.9; 17.3)	0.22 (0.13; 0.35)	< 0.001	0.217
	Maternal height [cm]	17.4 (10.6; 24.1)	0.23 (0.14; 0.32)	< 0.001	
	Female neonate yes/no	− 137.8 (− 215.1; −60.5)	−0.6 (−0.94; − 0.26)	0.001	
	Gestational weight gain [kg]	10.4 (3.4; 17.4)	0.136 (0.04; 0.23)	0.004	
	75 g OGTT fasting [mg/dL]	4.8 (1.1; 8.6)	0.12 (0.63; 4.9)	0.011	
Birth weight – *for a subgroup with a history of at least one delivery*	Birth weight of the largest child from previous pregnancy/ pregnancies [g]	0.26 (0.17; 0.35)	0.35 (0.23; 0.47)	< 0.001	0.358
	Gestational weight gain [kg]	13.9 (3.9; 24.0)	0.17 (0.05; 0.32)	0.007	
	Female neonate yes/no	−202.9 (− 318.0; −87.8)	−0.21 (−0.33; −0.09)	0.001	
	Maternal abdominal circumference [kg]	11.6 (4.0; 19.2)	0.198 (0.07; 0.33)	0.003	
	Maternal height [cm]	10.4 (0.4; 20.4)	0.128 (0.05; 0.25)	0.041	
Ponderal Index [g/cm^3^]	Birth weight [g]	0.001 (0.001; 0.002)	0.20 (0.20; 0.40)	< 0.001	0.039

*CI* Confidence interval, *OGTT* Oral Glucose Tolerance Test, *BMI* Body Mass Index, *GDM* Gestational Diabetes Mellitus

A ROC analysis was used to calculate the diagnostic power of maternal abdominal circumference measured before delivery as a predictor of adverse perinatal outcomes in the entire cohort (GDM patients and the control group) (Table 5).

**Table 5:** ROC curve analysis for maternal abdominal circumference measured prior the delivery as a predictor of selected maternal and foetal outcomes in the study group

The outcome	AUC (95% CI)	*P*	Cut-off value	Sensitivity	Specificity	OR (95% CI) for the outcome at the cut-off
Composite adverse maternal outcome	0.65 (0.61; 0.70)	< 0.001	100 cm	74.4%	49.6%	1.38 (1.22; 1.57)
Macrosomia	0.63 (0.58; 0.68)	0.0041	98 cm	91.4%	32.4%	1.24 (1.12; 1.37)
Neonatal hyperbilirubinemia	0.58 (0.53; 0.63)	0.033	108 cm	35.5%	79.0%	1.46 (1.02; 2.09)
Neonatal hypoglycaemia in the group with GDM	0.80 (0.74; 0.85)	< 0.0001	103 cm	88.9%	60.3%	2.01 (1.52; 2.64)
Rare adverse neonatal outcome	0.64	0.004	104 cm	60.0%	63.5%	1.53 (1.14; 2.06)

*ROC* Receiver Operating Characteristic, *AUC* Area Under the Curve, *CI* Confidence Interval, *OR* Odds Risk

Two-sided *p <* 0.05 was considered statistically significant.

Ethics approval for this study was obtained from the Warsaw Medical University institutional review board (AKBE13/15).

## Results

GDM was diagnosed on average during 25,3 +/− 1,5 weeks of pregnancy (between 24 and 28 weeks). The average time of diabetes-specific counseling was at 28,5 +/− 2,6 weeks and the mean time from diagnosis to treatment was 3,1 +/− 2,3 weeks (between 0 and 13 weeks). Average time of the delivery in GDM cohort was 38,8 weeks (between 37 and 42 weeks) and mean time from GDM recognition to delivery was 13,5 +/− 1,8 weeks (between 9 and 18 weeks).

Women with GDM compared to women without GDM had significantly older mean age (32.4 ± 4.7 vs 31.1 ± 4.1, *p* < 0.001) with significantly higher mean BMI before pregnancy (24.6 ± 4.6 vs 22.7 ± 3.6, p < 0.001), more frequently were obese (13.5% vs 3%, *p* < 0.001), were less likely to give birth for the first time (61,8% vs 49,5%, *p* < 0.024), and had a history of GDM-complicated pregnancy (18.8% vs 0%, p < 0.001). The results are shown in Table 1.

Pregnant women diagnosed with GDM gained significantly less weight during pregnancy (10.7 kg ± 5.8 vs 14.5 kg ± 5.1, p < 0.001) [Table 1], and had less frequent excess weight gain according to IOM criteria (24.2% vs 47.5%); instead, weight gain below the recommended IOM guidelines was more frequently observed in this group (Table 2).

No statistically significant differences were observed between the groups of patients with GDM and without GDM with respect to the incidence of maternal complications (including pregnancy induced hypertension or preeclampsia, mode of the delivery, intrapartum injury or haemorrhage), the condition of new-borns or the incidence of neonatal complications (birth weight > 4000 g, neonatal hypoglycaemia or hyperbilirubinemia, congenital malformation or other rare composite adverse outcome).

There was only a trend for borderline statistical significance of more frequent urgent caesarean sections due to foetal indications in the group with gestational diabetes (26.4% vs 16.2%, *p* = 0.06). The results are shown in Table 2.

Tables 3 and 4 show the predictive factors for individual perinatal complications in the entire study group. The results indicate that both the diagnosis of GDM, as well as maternal BMI prior to pregnancy, significantly modified the risk of excessive and insufficient weight gain during pregnancy. The importance of the female sex of the foetus as a factor significantly increasing the probability of insufficient weight gain during pregnancy requires underlying [OR: 1.65 (1.10; 2.47)].

On the other hand, the only parameters significantly influencing the risk of composite adverse maternal outcome were the maternal abdominal circumference [OR: 1.08 (1.04; 1.11)] and multiparity, which reduced the risk by almost half [OR: 0.47 (0.30; 0.75). The analysis of predictors of neonatal complications indicated that the abdominal circumference of the pregnant woman significantly increased the risk of all examined endpoints.

Analysis of the standardized coefficients summarised in Tables 3 and 4 indicates that maternal characteristics easily available from maternal history, like multiparity, maximum birth weight of a previous child (for women who delivered at least once), have a substantial impact on several outcomes. A GDM status is an important predictor for maternal outcomes related to the gestational weight gain. While maternal BMI prior to pregnancy was a statistically significant predictor for several foetal and maternal complications, standardized coefficients indicate a very small impact on these outcomes. This observation applies also for the maternal abdominal circumference measured prior to the delivery. Except from insulin therapy status which emerged both as a significant predictor and important contributing factor for a phototherapy in the GDM arm of the study, markers of maternal glycaemic status measured during the 75 g OGTT did not have a measurable impact on the outcomes, for which they were found to be statistically significant predictors.

The size of the maternal abdominal circumference before delivery was a strong factor correlating with the occurrence of perinatal complications in both the mother and the foetus in the entire cohort (Table 5). A circumference over 100 cm increased the risk of composite adverse maternal outcome by almost 40% (OR 1.38, *p* < 0.001). A circumference over 98 cm increased the risk of foetal macrosomia by 20% (OR 1.24, *p* < 0.005), and a circumference over 103 cm increased the risk of any neonatal complications by 50% (OR 1.54, p < 0.005). Furthermore, in the group with GDM, a circumference over 103 cm doubled the risk of neonatal hypoglycaemia during the first days of life (OR 2.01, *p* < 0.0001).

## Discussion

The number of patients with gestational diabetes in the world is continuously increasing. According to various estimates, over the last 20 years, the percentage of women with GDM has increased by 10–100%, especially in highly developed countries, and in 2019, hyperglycaemia was diagnosed in approximately 16% of pregnancies worldwide, of which GDM accounted for 84% of all cases [3, 18–22]. This fact allows us to predict a significant increase in the number of perinatal complications and forces researchers to identify factors that may affect their development and patients’ costs to eliminate them.

In our study, we demonstrated that the diagnosis of diabetes in pregnancy increases the risk of having a child with macrosomia by 10-fold (OR 10.4, *p* < 0.005) and 13-fold in multiparous women (OR 13.9, *p* < 0.005). These results are identical to other available publications [23–26]. To date, the individual influence of blood glucose values at individual measurement points in the 75-g OGTT on obstetric complications is not fully understood. The HAPO study, which was the basis for changing the existing criteria for the diagnosis of GDM, demonstrated a linear relationship between maternal glucose levels and the child’s birth weight [10]. Zhu et al. and Zawiejska et al. found that macrosomia was diagnosed significantly more often in children of patients with fasting hyperglycaemia [20, 27]. On the other hand, Kerenyi et al. [26] found that the curve illustrating the relationship between fasting glucose measured during the 75 g OGTT and the neonatal birth weight and the risk of LGA was U-shaped (*p* = 0.004), indicating that both in patients with low and high fasting blood glucose levels, the risk of foetal hypertrophy was increased. In a publication by Black et al. [28], attention was also drawn to the significant influence of hyperglycaemia at 2 hrs 75 g OGTT on the increased risk of pregnancy induced hypertension (PIH), preterm labour and hyperbilirubinemia in new-borns. Despite these findings, our study did not identify any correlation between the glycaemic status of patients from particular groups and the percentage of maternal (here: pre-eclampsia) or foetal complications (Table 2). We therefore feel this issue leaves area for further research.

On the other hand, we found significant predictors of perinatal complications, independent of the severity of hyperglycaemia at the time of GDM diagnosis, included patients’ anthropometric markers related to the amount of adipose tissue, i.e., BMI before pregnancy and abdominal circumference measured before delivery. Many studies have confirmed that overweight and obesity before pregnancy are independent risk factors for the development of perinatal complications [29–35]. In the multicentre LifeCycle Project-Maternal Obesity and Childhood Outcomes Study group, maternal and foetal complications were observed in as many as 61% of pregnancies in women with a BMI ≥40 kg/m^2^ [35]. Ouzounian et al. determined that the risk of macrosomia was doubled in patients with BMI ≥30 kg/m^2^ compared to pregnant women with normal BMI before pregnancy and was threefold higher in patients with excessive vs normal weight gain in pregnancy according to the IOM 2009 recommendations [29]. Similar relationships were demonstrated in the work of Bodnar et al. [36]. In our study, we found that higher pre-pregnancy BMI values correlated with a higher risk of pre-eclampsia during pregnancy (OR 1.15, *p* < 0.001). On the other hand, the increased abdominal circumference measured in patients before delivery had a significant impact on increasing risk of perinatal complications in women (increased blood loss, injury of soft tissues of the birth canal) (OR 1.08, *p* < 001), emergency caesarean section due to foetal risk (8% increase) and high birth weight of new-borns (12-fold increase in the risk; OR 12.1, *p* < 0.001). Moreover, among other complications during the early neonatal period, we observed influence of high maternal abdominal circumference on increasing risk of hypoglycaemia in the first days of life (by 12%; OR 1.12, *p* < 0.001), the need for phototherapy due to hyperbilirubinemia (increase by 7%) and the risk of at least one complication during the neonatal period, i.e., hyperbilirubinemia, hypoglycaemia or rare complications (by 8%; OR 1.08, *p* < 0.001). Interestingly, we noticed that there was no difference in maternal abdominal circumference between women with GDM and their normoglycemic counterparts. However, when we looked for predictors for perinatal complications in the whole cohort we thought out that maternal abdominal circumference is a significant predictor for these complications. It means that there was no difference in maternal abdominal circumference regarding the exposure but this parameter was a significant predictor for the outcome. These results are consistent with other available publication [37].

It is also worth emphasizing that in the population of non-pregnant women, waist circumference is considered an indicator of insulin resistance, and its increased value has been included in the criteria for diagnosing metabolic syndrome [38–42]. Some sources report waist-to-hip ratio (WHR) to be superior to BMI for predicting the risk of developing type 2 diabetes, hypertension and cardiovascular disease in adults [43–47]. Obviously, the measurement of abdominal circumference in pregnancy is a specific measurement that is technically difficult and related to a non-standard population, but the relationships observed in our study between neonatal complications and increased abdominal circumference in term pregnancy confirm that this parameter also informs about the “metabolic condition” of the mother and should be taken into account in the context of expected perinatal complications. Our observation of the influence of maternal parameters related to insulin resistance on the risk of obstetric complications may also explain the persistence of a high percentage of perinatal complications in the population of pregnant women with hyperglycaemia in pregnancy, despite optimization of metabolic control. This was also confirmed by data from our multivariate regression models, which indicate that weight gain during pregnancy and the circumference of the pregnant woman’s abdomen measured before delivery, and not the severity of carbohydrate tolerance disorders, remain risk factors for significant obstetric complications. This means that the parameters describing the “maternal metabolic status” remain a significant risk factor for adverse maternal-foetal outcomes when effective treatment eliminates the risk associated with hyperglycaemia in pregnancy. Therefore, we suggest that both traditional risk factors and new biomarkers should be taken into account when prognosing adverse perinatal outcomes.

Aforementioned risk factor for perinatal complications includes excessive weight gain during pregnancy [29–31, 48]. GWG consistent with the Institute of Medicine guidelines is associated with better maternal and neonatal outcomes. However, while appropriate gain is highly desirable, it is achieved in only a minority of pregnancies. Kominiarek et al. found that GWG above that recommended ranges was associated with an increased risk of shoulder dystocia (OR 1.74, 95% CI 1.41–2.14), macrosomia (OR 2.66, 95% CI 2.03–3.48) and neonatal hypoglycaemia (OR 1.60, 95% CI 1.16–2.22) [49]. In a systematic review and meta-analysis by Goldstein et al., excessive weight gain during pregnancy correlated with a significantly higher rate of caesarean sections (OR 1.30, 95% CI 1.25–1.35), macrosomia (OR 1.95, 95% CI 1.79–2.11) and LGA (OR 1.85, 95% CI 1.76–1.95). Interestingly, in the same study, it was noted that if the weight gain during pregnancy was too low, it significantly increased the risk of small for gestational age (SGA) baby (OR 1.53, 95% CI 1.44–1.64) and preterm labour (OR 1.70, 95% CI 1.32–2.20) and decreased the risk of LGA and macrosomia [50]. Similar results were found in the work of Papazian et al. [51]. In our study, we did not identify any significant relationships between weight gain in pregnant women and the risk of perinatal complications what refers to the IOM report noted there was a lack of evidence regarding the role of GWG in relation to GDM [52]. On the other hand, LifeCycle Project-Maternal Obesity and Childhood Outcomes Study group findings suggest that pre-pregnancy weight might be a more important target for interventions than gestational weight gain [35]. This suggests that the use of IOM guidelines may need to be reconsidered for individual prediction of the risk for adverse pregnancy outcomes.

We did notice that patients with GDM, compared to healthy pregnant women, had a significantly higher pre-pregnancy BMI (24.6 ± 4.6 vs 22.7 ± 3.6, *p* < 0.01) and were more frequently obese (13.5% vs 3%, *p* < 0.001). The diagnosis of diabetes in pregnancy was associated with a twice lower risk of excessive weight gain during pregnancy (OR 2.43, *p* = 0.001), although the increased risk of excessive weight gain was significantly associated with a higher BMI before pregnancy (OR 1.12, *p* < 0.001), higher fasting glucose (1.03, *p* < 0.05) and lower 2 hrs glucose during 75 g OGTT (OR 0.99, *p* < 0.05). Fasting hyperglycaemia is a marker of hepatic insulin resistance and one of the components of metabolic syndrome [53]. Therefore, in the context of our research, the positive relationship between excessive weight gain during pregnancy and fasting hyperglycaemia should be interpreted as a clinical manifestation of the relationship between pregnancy weight gain and insulin resistance.

As we calculated, the diagnosis of diabetes during pregnancy reduced the risk of excessive weight gain in pregnancy by 30% (OR 0.31, *p* < 0.001) and increased the risk of weight gain under 12 kg (OR 4.44, *p* < 0.01) by four-fold. These results also suggest that treatment of hyperglycaemia with well-controlled diabetes in pregnancies that still experience complications result from non-glycaemic risk factors, including components of metabolic syndrome. One of the effects of multidisciplinary care for pregnant women with GDM and at the same time the only cost of reducing adverse outcomes found in our research may be lowering weight gain during pregnancy, which is difficult to interpret unequivocally due to the lack of guidelines for the group of patients with GDM. For others, it is possible to slow down intrauterine growth, which protects against macrosomia and intrauterine death of the foetus. In the light of the data available to us, it seems that the price of these benefits may be an increased risk of accelerated weight gain in infants and obesity in early school age children [54, 55]. However, one should also take into account the latest data presented by the LifeCycle Project consortium, which showed, in a population of approximately 200,000 pregnant women, that in women with a BMI > 30, the optimal weight gain for reducing obstetric complications is lower (0–6 kg) than that recommended by the IOM for pregnant women with a similar BMI (5–9 kg) [35]. Additionally, authors of a retrospective observational study of 2842 women with GDM published in 2020 confirmed the dominant pattern of gestational weight gain below the level recommended by IOM in this population (50.3% of the examined patients) [52]. Future research should assess whether optimal GWG ranges proposed by IOM in combination with other maternal and foetal pregnancy characteristics are useful for prediction of adverse pregnancy outcomes.

The results of our study indicate that the glycaemic status of patients may be a predictor of certain maternal complications, including abnormal weight gain during pregnancy, but neither gestational diabetes nor blood glucose levels at individual 75 g OGTT measurement points were predictors of neonatal complications in the study cohort. On the other hand, it is noteworthy that the anthropometric conditions of pregnant women and gestational weight gain, which are indicators of the “metabolic status” of women, may significantly correlate with the occurrence of obstetric complications.

At this point we must acknowledge some limitations of our study. First, it was a single centre and examined only short-term outcomes. Probably long-term multicentre study may show more benefit. Second, main finding of lower gestational weight gain in women with GDM was based on a self-reported maternal pre-pregnancy weight and only the last noted value was measured. This may have led to misclassification of GWG. In the future we plan to include to the assessment of women’s ‘metabolic status’ other measurements like percentage of adipose tissue.

Strengths of our study include the robustness of the results after several statistical analyses. What is more, to assess adverse pregnancy outcomes in the entire cohort we compared patients with GDM and non-GDM who would have been diagnosed regardless of the adopted criteria for the diagnosis of diabetes (either according to the 2011 or 2014 criteria), what seems to be an appropriate denominator for the results. We also proved that treatment of mild hyperglycaemia in pregnancy results in a normalization of getting perinatal outcomes similar with those from normoglycemic population. However, then we have seen that the risk factor which remains is related to maternal hyperinsulinemia which is more complex than hyperglycaemia alone and may be a source of complications in a population of normoglycemic one. This observation may be very important considering that the hyperglycaemia is a continued risk factor for maternal complications and all thresholds set up for hyperglycaemia are arbitrary. Therefore, in our study, we have provided the evidence for another targets for therapy which should be addressed to women planning a pregnancy. We also indicated that measurement of this parameter is useful if we achieve normoglycaemia in women treated for GDM. Our study also provided explanation why despite of the success of hyperglycaemia treatment maternal diabesity is still related to perinatal complications.

## Conclusions

No differences were found in maternal and foetal outcomes in GDM and non-GDM women except gestational weight gain below Institute of Medicine recommendations. The only “cost” of reducing adverse pregnancy outcomes in GDM patients seems to be lowering gestational weight gain, the future impact of which on GDM pregnant population should be assessed. The maternal abdominal circumference measured before delivery not the severity of carbohydrate intolerance, remained the main predictor for significant perinatal complications.

## Data Availability

The datasets used or analysed during the current study are available from the corresponding author on reasonable request. Contact: anetapolubiec@interia.eu.

## Abbreviations

GDM: Gestational diabetes mellitus
IADPSG: The International Association of Diabetes and Pregnancy Study Groups
WHO: World Health Organization
OGTT: Oral glucose tolerance test
LGA: Large for gestational age
OR: Odds risk
IDF: International Diabetes Federation
HAPO: Hyperglycemia and Adverse Pregnancy Outcome
BMI: Body mass index
GWG: Gestational weight gain
aORs: Adjusted odds risk
CIs: Confidence intervals
ROC: Receiver operating characteristic
IOM: The Institute of Medicine
PIH: Pregnancy induced hypertension
WHR: Waist-to-hip ratio
SGA: Small for gestational age

## References

[1] Dukler D, Porath A, Bashiri A, Erez O, Mazor M (2001). Remote prognosis of primiparous women with preeclampsia. Eur J Obstet Gynecol Reprod Biol.

[2] Bellamy L, Casas JP, Hingorani AD, Williams D (2009). Type 2 dibetes mellitus after gestational diabetes: a systematic review and meta-analysis. Lancet.

[3] Gilbert JS, Banek CT, Babcock SA, Dreyer HC (2013). Diabetes in early pregnancy: getting to the heart of the matter. Diabetes.

[4] Damm P, Houshmand-Oeregaard A, Kelstrup L, Lauenborg J, Mathiesen ER, Clausen TD (2016). Gestational diabetes mellitus and long-term consequences for mother and offspring: a view from Denmark. Diabetologia.

[5] IDF Diabetes Atlas 10th Edition 2021. https://diabetesatlas.org/atlas/tenth-edition/. Accessed 15 March 2022.

[6] Wierzba W, Śliwczyński A, Karnafel W, Bojar I, Pinkas J (2017). Gestational diabetes mellitus/hyperglycaemia during pregnancy in Poland in the years 2010–2012 based on the data from the National Health Fund. Ginekol Pol.

[7] Getahun D, Nath C, Ananth CV, Chavez MR, Smulian JC (2008). Gestational diabetes in the United States: temporal trends 1989 through 2004. Am J Obstet Gynecol.

[8] Metzger BE, Gabbe SG, Persson B, Buchanan TA, Catalano PA, Damm P, Dyer AR, Ad L, Hod M, Kitzmiler JL, Lowe LP, HD MI, Oats JJ, Omori Y, Schmidt MI, International Association of Diabetes and Pregnancy Study Groups Consensus Panel (2010). International association of diabetes and pregnancy study groups recommendations on the diagnosis and classification of hyperglycemia in pregnancy. Diabetes Care.

[9] Diagnostic criteria and classification of hyperglycaemia first detected in pregnancy. World Health Organization 2013. http://apps.who.int/iris/bitstream/10665/85975/1/WHO_NMH_MND_13.2_eng.pdf. Accessed 18 Sept 2015. 24199271

[10] Metzger BE, Lowe LP, Dyer AR, Trimble ER, Chaovarindr U, Coustan DR, Hadden DR, McCance DR, Hod M, McIntyre HD, Oats JJ, Persson B, Rogers MS, Sacks DA, HAPO Study Cooperative Research Group (2008). Hyperglycemia and adverse pregnancy outcomes. N Engl J Med.

[11] Crowther CA, Hiller JE, Moss JR, McPhee AJ, Jeffries WS, Robinson JS (2005). Australian carbohydrate intolerance study in pregnant women (ACHOIS) trial group. Effect of treatment of gestational diabetes mellitus on pregnancy outcomes. N Engl J Med.

[12] Landon MB, Spong CY, Thom E, Carpenter MW, Ramin SM, Casey B, Wapner RJ, Varner MW, Rouse DJ, Thorp JM, Sciscione A, Catalano P, Harper M, Saade G, Lain KY, Sorokin Y, Peaceman AM, Tolosa JE, Anderson GB (2009). Eunice Kennedy Shriver National Institute of Child Health and Human Development maternal-fetal medicine units network. A multicenter, randomized trial of treatment for mild gestational diabetes. N Engl J Med.

[13] Hartling L, Dryden DM, Guthrie A, Muise M, Vandermeer B, Donovan L (2013). Benefits and harms of treating gestational diabetes mellitus: a systematic review and metaanalysis for the U.S. preventive services task force and the National Institutes of Health Office of medical applications of research. Ann Intern Med.

[14] Mei-Fang L, Li M, Yu TP, Zhu Y, Chen MY, Liu Y, Li LX (2020). Adverse maternal and neonatal outcomes in pregnant women with abnormal glucose metabolism. Diabetes Res Clin Pract.

[15] Blickstein I, Doyev R, Trojner Bregar A, Bržan Šimenc G, Verdenik I, Tul N (2018). The effect of gestational diabetes, pre-gravid maternal obesity, and their combination ('diabesity') on outcomes of singleton gestations. J Matern Fetal Neonatal Med.

[16] ADA (2013). Standards of medical care in diabetes. Diabetes Care.

[17] Standardy Polskiego Towarzystwa Ginekologicznego (2014). postępowanie u kobiet z cukrzycą – aktualizacja. Ginekol Pol.

[18] Hod M, Kapur A, Sacks DA, Hadar E, Agarwal M, Di Renzo GC, Cabero Roura L, McIntyre HD, Morris JL, Divakar H (2015). The International Federation of Gynecology and Obstetrics (FIGO) initiative on gestational diabetes mellitus: a pragmatic guide for diagnosis, management, and care. Int J Gynaecol Obstet.

[19] Wielgoś M, Bomba-Opoń D, Czajkowski K, Wender-Ożegowska E, Hod M (2017). Towards a European consensus on gestational diabetes mellitus: a pragmatic guide for diagnosis, management, and care. The polish diabetes in pregnancy study group and FIGO. Ginekol Pol.

[20] Zhu WW, Fan L, Yang HX, Kong LY, Su SP, Wang ZL, Hu YL, Zhang MH, Sun LZ, Mi Y, Du XP, Zhang H, Wang YH, Huang YP, Zhong LR, Wu HR, Li N, Wang YF, Kapur A (2013). Fasting plasma glucose at 24-28 weeks to screen for gestational diabetes mellitus: new evidence from China. Diabetes Care.

[21] Dabelea D, Snell-Bergeon JK, Hartsfield CL, Bischoff KJ, Hamman RF, McDuffie RS (2005). Kaiser Permanente of Colorado GDM screening program. Increasing prevalence of gestational diabetes mellitus (GDM) over time and by birth cohort: Kaiser Permanente of Colorado GDM screening program. Diabetes Care.

[22] IDF Diabetes Atlas Ninth Edition 2019. https://www.idf.org/our-activities/care-prevention/gdm. Accessed 7 Aug 2020.

[23] Yang J, Cummings EA, O'Connell C, Jangaard K (2006). Fetal and neonatal outcomes of diabetic pregnancies. Obstet Gynecol.

[24] Chatfield J (2001). ACOG issues guidelines on fetal macrosomia. American College of Obstetricians and Gynecologists. Am Fam Physician.

[25] Rockhill K, Dorfman H, Srinath M, Hogue C (2015). The effects of prepregnancy body mass index and gestational weight gain on fetal macrosomia among American Indian/Alaska native women. Matern Child Health J.

[26] Kerényi Z, Tamás G, Kivimäki M, Péterfalvi A, Madarász E, Bosnyák Z, Tabák AG (2009). Maternal glycemia and risk of large-for-gestational-age babies in a population-based screening. Diabetes Care.

[27] Zawiejska A, Wender-Ozegowska E, Radzicka S, Brazert J (2014). Maternal hyperglycemia according to IADPSG criteria as a predictor of perinatal complications in women with gestational diabetes: a retrospective observational study. J Matern Fetal Neonatal Med.

[28] Black MH, Sacks DA, Xiang AH, Lawrence JM (2010). Clinical outcomes of pregnancies complicated by mild gestational diabetes mellitus differ by combinations of abnormal oral glucose tolerance test values. Diabetes Care.

[29] Ouzounian JG, Hernandez GD, Korst LM, Montoro MM, Battista LR, Walden CL, Lee RH (2011). Pre-pregnancy weight and excess weight gain are risk factors for macrosomia in women with gestational diabetes. J Perinatol.

[30] Heude B, Thiebaugeorges O, Goua V, Forhan A, Kaminski A, Foliguet B, Schweitzer M, Magnin G, Charles MA (2012). Pre-pregnancy body mass index and weight gain during pregnancy: relations with gestational diabetes and hypertension, and birth outcomes. Matern Child Health J.

[31] Bowers K, Laughon SK, Kiely M, Brite J, Chen Z, Zhang C (2013). Gestational diabetes, pre-pregnancy obesity and pregnancy weight gain in relation to excess fetal growth: variations by race/ethnicity. Diabetologia.

[32] Castro LC, Avina RL (2002). Maternal obesity and pregnancy outcomes. Curr Opin Obstet Gynecol.

[33] Yu CK, Teoh TG, Robinson S (2006). Obesity in pregnancy. BJOG.

[34] Raatikainen K, Heiskanen N, Heinonen S (2006). Transition from overweight to obesity worsens pregnancy outcome in a BMI-dependent manner. Obesity.

[35] LifeCycle Project- Maternal Obesity and Childhood Outcomes Study Group (2019). Association of gestational weight gain with adverse maternal and infant outcomes. JAMA.

[36] Bodnar LM, Siega-Riz AM, Simhan HN, Himes KP, Abrams B (2010). Severe obesity, gestational weight gain, and adverse birth outcomes. Am J Clin Nutr.

[37] Gao X, Yan Y, Xiang S, Zeng G, Liu S, Sha T (2017). The mutual effect of pre-pregnancy body mass index, waist circumference and gestational weight gain on obesity-related adverse pregnancy outcomes: a birth cohort study. Plos One.

[38] Lee S, Bacha F, Gungor N, Arslanian SA (2006). Waist circumference is an independent predictor of insulin resistance in black and white youths. J Pediatr.

[39] Messiah SE, Arheart KL, Lipshultz SE (2008). Body mass index, waist circumference, and cardiovascular risk factors in adolescents. J Pediatr.

[40] Expert Panel on Detection, Evaluation, and Treatment of High Blood Cholesterol in Adults. Executive Summary of the Third Report of the National Cholesterol Education Program (NCEP) Expert Panel on Detection, Evaluation, and Treatment of High Blood Cholesterol in Adults (Adult Treatment Panel III). JAMA. 2001;285(19):2486–97. 10.1001/jama.285.19.2486. 10.1001/jama.285.19.2486 11368702

[41] Zimmet P, Magliano D, Matsuzawa Y, Alberti G, Shaw J (2005). The metabolic syndrome: a global public health problem and a new definition. J Atheroscler Thromb.

[42] Grundy SM, Cleeman JI, Daniels SR, Donato KA, Eckel RH, Franklin BA, Gordon DJ, Krauss RM, Savage PJ, Smith SC (2005). Diagnosis and management of the metabolic syndrome: an American Heart Association/National Heart, Lung, and Blood Institute Scientific Statement. Circulation.

[43] Taebi M, Sadat Z, Saberi F, Kalahroudi MA (2015). Early pregnancy waist-to-hip ratio and risk of preeclampsia: a prospective cohort study. Hypertens Res.

[44] Cheng CH, Ho CC, Yang CF, Huang YC, Lai CH, Liaw YP (2010). Waist-to-hip ratio is a better anthropometric index than body mass index for predicting the risk of type 2 diabetes in Taiwanese population. Nutr Res.

[45] Oliveira M, Fagundes R, Moreira E, Trindade E, Carvalho T (2010). Relation between anthropometric indicators and risk factors for cardiovascular disease. Arq Bras Cardiol.

[46] Winter Y, Rohrmann S, Linseisen J, Lanczik O, Ringleb PA, Hebebrand J, Back T (2008). Contribution of obesity and abdominal fat mass to risk of stroke and transient ischemic attacks. Stroke.

[47] Feldstein CA, Akopian M, Olivieri AO, Kramer AP, Nasi M, Garrido D (2005). A comparison of body mass index and waist-to-hip ratio as indicators of hypertension risk in an urban argentine population: a hospital-based study. Nutr Metab Cardiovasc Dis.

[48] Rasmussen KM, Yaktine AL, Institute of Medicine (US) and National Research Council (US) Committee to Reexamine IOM Pregnancy Weight Guidelines (2009). Weight Gain During Pregnancy: Reexamining the Guidelines.

[49] Kominiarek MA, Saade G, Mele L, Bailit J, Reddy UM, Wapner RJ, Varner MW, Thorp JM, Caritis SN, Prasad M, Tita ATN, Sorokin Y, Rouse DJ, Blackwell SC, Tolosa JE (2018). Eunice Kennedy Shriver National Institute of Child Health and Human Development (NICHD) maternal-fetal medicine units (MFMU) network. Association between gestational weight gain and perinatal outcomes. Obstet Gynecol.

[50] Goldstein RF, Abell SK, Ranasinha S, Misso M, Boyle JA, Black MH, Li N, Hu G, Corrado F, Rode L, Kim YJ, Haugen M, Song WO, Kim MH, Bogaerts A, Devlieger R, Chung JH, Teede HJ (2017). Association of Gestational Weight Gain with Maternal and Infant Outcomes: a systematic review and Meta-analysis. JAMA.

[51] Papazian T, Abi Tayeh G, Sibai D, Hout H, Melki I, Rabbaa KL (2017). Impact of maternal body mass index and gestational weight gain on neonatal outcomes among healthy middle-eastern females. Plos One.

[52] Xie X, Liu J, Pujol I (2020). Inadequate weight gain according to the Institute of Medicine 2009 guidelines in women with gestational diabetes: frequency, clinical predictors, and the association with pregnancy outcomes. J Clin Med.

[53] Huang PL (2009). A comprehensive definition for metabolic syndrome. Dis Model Mech.

[54] Matthews EK, Wei J, Cunningham SA (2017). Relationship between prenatal growth, postnatal growth and childhood obesity: a review. Eur J Clin Nutr.

[55] Tzoulaki I, Sovio U, Pillas D, Hartikainen AL, Pouta A, Laitinen J, Tammelin TH, Jarvelin MR, Elliott P (2010). Relation of immediate postnatal growth with obesity and related metabolic risk factors in adulthood: the northern Finland birth cohort 1966 study. Am J Epidemiol.

